# Impact on child vaccination completion rates of short message services (SMS) reminders in developing countries

**DOI:** 10.11604/pamj.supp.2020.35.1.19442

**Published:** 2020-02-18

**Authors:** Robert Davis

**Affiliations:** 1 American Red Cross, Nairobi, Kenya

**Keywords:** Measles, routine immunization, recall and reminder systems, electronic immunization registers

## Abstract

**Introduction:**

The Expanded Programme on Immunization has, since its inception, struggled to achieve high completion rates for child immunizations. The introduction of 2YL (second year of life) immunizations presents the programme with fresh challenges to assuring high completion rates.

**Methods:**

Using the same procedures as those employed in the 2017 article on SMS reminders, of which this is an update, I searched the NLM database for all recent articles from developing countries on SMS reminders for reduction of vaccination dropout rates. I summarized these and earlier articles in tabular form.

**Results:**

The freshly reviewed articles are confirmatory of earlier studies which show an improvement in vaccination completion rates when SMS reminders are sent to mothers and other caregivers.

**Conclusion:**

All of the studies reviewed were based on pilot projects. It is time, and past time, to go to scale with SMS reminders, perhaps stand alone, or as part of a larger system of electronic immunization registers. There may be potential for use of WhatsApp in dropout reduction, thus far documented only in other public health applications.

## Introduction

When the Expanded Programme on Immunization was created in 1974, there were six diseases targeted for infant vaccination. Vaccinations began at birth and were completed with measles vaccine, which was typically given from 9 months of age. In the current century, most vaccination programmes vaccinate against a dozen or more childhood diseases, and many have gone over to a second year of life (2YL) delivery platform, including a second dose of measles containing vaccine given from 15 or 18 months of life. Although, by 2017, there were 167 countries implementing MCV2 in the second year of life, dropouts between the first and the second dose remained a problem (Table 1). Since measles vaccination at 9 months confers only about 85 percent protection, the failure to complete the MCV series is an important obstacle to the measles elimination goal endorsed by all six of the W.H.O. regions. Table 1 shows the heterogeneity of coverage statistics. The Republic of Rwanda already has coverage statistics consistent with interruption of measles transmission. Nigeria has coverage for all doses of all antigens which require improvement. Kenya, Senegal and Zimbabwe all show coverage and dropout figures which would benefit from reminder systems. It is vitally important, both for measles eradication and for protection against other vaccine preventable diseases, that all multi-dose vaccinations be complete and on time. A 2018 Cochrane review [1] examined evidence on reminder and recall systems for assuring completion of vaccinations from both developed and developing countries. The authors concluded that “patient reminder and recall systems, in primary care systems, are likely to be effective at improving the proportion of the target populations who receive immunizations.”

**Table 1:** WHO/UNICEF estimates of vaccination coverage, selected African countries, 2017

Country	DPT 1	MCV1	MCV2
Ethiopia	85	65	N/A
Kenya	93	89	35
Madagascar	80	58	N/A
Mozambique	90	85	45
Nigeria	49	42	N/A
Rwanda	99	95	95
Senegal	97	90	70
South Africa	74	66	60
Uganda	95	80	N/A
Zimbabwe	94	90	78

Source: World Health Organization Vaccine-preventable diseases: Monitoring System, 2018 Global Summary, consulted on 13 May 2019

## Methods

The present article is an update of a 2017 article, published in this journal, on the impact of SMS reminders on child vaccination completion rates, with specific reference to sub-Saharan Africa [2]. We have followed the methodology of Manakongtreecheep, described in his article, and have included studies published since 2017 and articles from outside Africa. The present review complements a systematic review by Mekonnen and colleagues, who found from their meta-analysis that SMS reminders had a significant impact on child vaccination coverage [3]. There are two from Kenya [4, 5], two from Nigeria [6,7] and one each from Zimbabwe [8], Burkina Faso [9], Guatemala [10], China [11], Bangladesh [12], India [13] and Pakistan [14]. Neither the current study, nor the much more comprehensive Cochrane update, has looked at nationwide SMS reminder systems.

## Results

The five additional studies, summarized in Table 2, have added to our understanding of SMS messaging. The Guatemala study is cautionary: when completion rates are already very high, the marginal benefit from SMS reminders may be less than in underperforming countries. The China study shows an additive impact of mobile phone app and texting, compared to the impact of the mobile phone app alone. The Indian study summarized in Table 2 shows the compliance linked group achieved higher results than the SMS reminders. This confirms the results of the first Kenyan study, which also showed a positive impact of cash incentives. Both these studies raise issues about long term financial sustainability of cash incentives. The Pakistan study showed a 10 percent difference in coverage, based on the per protocol analysis, confirming the positive results of the studies reviewed in 2017. The Bangladesh study is also confirmatory of the earlier African studies, showing significant coverage improvements associated with SMS reminders.

**Table 2:** Published findings on impact of SMS Messaging on vaccination dropouts, developing countries

Country	Author(s)	Main findings
Kenya	Gibson *et al.*	Vaccination coverage ↑ 4% with SMS, ↑8% with SMS plus conditional cash transfer
Kenya	Haji *et al*.	Dropouts were 4 % among SMS recipients, 17% among controls.
Nigeria	Brown *et al*.	Ibadan mothers willing to record their numbers at clinics for reminder/recall, in preference to home visits and Email reminders.
Nigeria	Eze, Adeleye	Coverage 8.7% higher in the intervention group; SMS reminders cheaper than house visits
Zimbabwe	Bangure *et al*.	Coverage at 14 weeks was 95% in the intervention group and 75% in the non-intervention group, p<0.001.
Burkina Faso	Schlumberger *et al.*	At 4 months of age, attendance for children was significantly better for children whose parents were sent SMS messages, p<0.001.
Guatemala	Domek *et al.*	“Both intervention and usual care participants had high rates of vaccine and visit completion, with a non- statistically significant higher percentage of children in the intervention completing both visit 2 and visit 3.”
China	Chen *et al.*	“An app and text messages can be used by village doctors to improve full vaccination coverage, though no significant increase in coverage was found when assessing the effect of the app on its own.”
Bangladesh	Uddin *et al.*	“Difference-in-difference estimates were +29.5% for rural intervention versus control areas and +27.1% for urban areas.”
India	Seth *et al.*	“Median coverage at enrolment was 33% in all groups and increased to 41.7%, 40.1% and 50.0% by the end of the study in the control group, the group with mobile phone reminders, and the compliance-linked incentives group, respectively.”
Pakistan	Kazi *et al.*	“Only children in the per protocol analyses, who received an SMS reminder for vaccine uptake at 6 weeks visit, showed a statistically significant difference (96.0% vs 86.4%).”

Note: The first six citations are from Manakongtreecheep, *op. cit*.

## Discussion

The studies reviewed showed a positive impact on routine vaccination coverage of SMS reminder systems. All were of pilot projects. Further work in this area is important as more countries move to a 2YL (second year of life) approach, with more demanding requirements for sustained high coverage over the first two years of life. Without improved MCV2 coverage, most developing countries (Rwanda is a remarkable exception) will continue to need measles campaigns every two or three years, with all which this implies in terms of demands on human resources at the national and subnational levels. It would be useful for governments and donors in countries with successful SMS projects to go to scale. Countries which have not yet launched SMS projects may wish to do so, especially if they have poor completion rates.

SMS reminders are but one of several approaches to vaccination reminders. The most recent Cochrane review, cited above, lists telephone calls, letters, postcards, text messages, and autodial messages as among reminder/recall methods. However, many of these rely, for example, on efficient postal systems for implementation. Some are labour intensive, whereas health workers in developing countries are often short of time. When part of a larger system including birth registration, SMS reminders have the potential to work without heavy time inputs and wherever mothers or other caregivers have access to mobile telephones. Combining SMS reminders with an electronic immunization register, as in Burkina Faso, places the SMS reminder in a larger, comprehensive health management information system. Such registers, in addition, present economic advantages in terms of savings on printing. Voice reminders, only feasible in places with stable network signals, are an alternative to SMS messaging.

## Conclusion

Like its 2017 forerunner, this review covers only pilot projects, since no pilots have been scaled up. For scale-up, both management capacity and costing need careful analysis. The costs of SMS messaging and of other methods need careful assessment. Will reminders using WhatsApp, not yet well documented, emerge as a less expensive reminder method? There may be potential for use of WhatsApp, but the 303 publications listed on WhatsApp in the National Library of Medicine (NLM) database (consulted at end October 2019) cover such topics as clinical medicine and, for developing countries, such activities as bed net use and smoking cessation. So WhatsApp use for immunization remains in posse rather than in esse. As of this writing (2019), SMS messaging is certainly among the best documented and most promising technologies for improving childhood vaccination completion rates.

### What is known about this topic

- No pilots review have been scaled up.

### What this study adds

- SMS messaging is certainly among the best documented and most promising technologies for improving childhood vaccination completion rates.

## Competing interests

The authors declare no competing interests.
